# Stress-Induced Cardiomyopathy Masquerading as Acute ST-Segment Elevation Myocardial Infarction

**DOI:** 10.7759/cureus.42181

**Published:** 2023-07-20

**Authors:** Subash Nepal, Dana Aiello, Kamala Ojha

**Affiliations:** 1 Internal Medicine, Upstate Medical University, Syracuse, USA; 2 Cardiovascular Disease, Upstate University Hospital, Syracuse, USA; 3 Internal Medicine, Upstate University Hospital, Syracuse, USA

**Keywords:** coronary artery angiogram, echocardiogram, apical ballooning syndrome, st-elevation myocardial infarction, stress-induced cardiomyopathy

## Abstract

Myocardial stress can lead to a myriad of cardiovascular complications, and stress-induced cardiomyopathy is the predominant manifestation. Exogenous or endogenous hormonal excess, sepsis, tachycardia, and physical or emotional trauma can lead to neurohormonal and catecholaminergic surges. Stress-induced cardiomyopathy often presents with chest pain, ischemic-like ECG changes, troponin elevation, and wall motion abnormalities in echocardiography. It is a diagnosis of exclusion, and coronary artery disease needs to be ruled out by a normal angiogram as per guidelines. It presents predominantly in postmenopausal women and presentation is similar to acute coronary syndrome (ACS) due to plaque rupture. We report a case of a 72-year-old female who presented to the emergency room with severe anginal chest pain without any preceding stress. ECG showed lateral leads ST-elevation and serial serum troponins were elevated. Emergent cardiac catheterization showed insignificant coronary artery disease. Left ventriculogram and echocardiogram showed a moderately reduced left ventricular systolic function with akinetic-hypokinetic mid to distal myocardial segments and normal basal contraction suggestive of stress-induced cardiomyopathy.

## Introduction

Acute myocardial stress can cause a myriad of cardiac manifestations. Stress-induced cardiomyopathy is the most common cardiac sequelae. It frequently masquerades as an acute coronary syndrome (ACS) but these patients do not have underlying atherosclerotic coronary artery disease. It commonly presents in postmenopausal women and is believed to be due to stress-induced neurohormonal surge, exposure to female sex hormones, and catecholaminergic toxicity leading to coronary microvascular dysfunction and apical stunning [1]. It is usually preceded by physical or emotional stress, although some cases lack an evident precipitant. Myocardial stress causes a catecholaminergic excess state, leading to hypokinesis of the cardiac apex and hypercontractility of the base, causing classical apical ballooning. It commonly presents with typical chest pain, ST-segment changes, and troponin elevation. Stress-induced cardiomyopathy is a diagnosis of exclusion, and an invasive coronary angiogram must be performed to rule out epicardial coronary artery disease. While it is generally reversible, systolic dysfunction may persist in some cases, leading to chronic non-ischemic cardiomyopathy. Stress causes a surge of adrenaline (Epi) from the adrenal medulla and noradrenaline (NE) from cardiac nerve endings due to excitation of the medullary autonomic system. Circulating adrenal Epi exerts stronger hormonal effects on the cardiac tissue than NE [2]. The basal myocardium is rich in sympathetic nerve terminals and NE, whereas the apex is rich in β1-receptors [3,4]. High NE thus results in vigorous basal contraction. However, excess Epi in the apex caused by low clearance due to sparse neural uptake leads to excitotoxicity of the apical adrenoreceptors and intracellular Ca 2+ overload [1,5]. This is compounded by an increased afterload state created by increased NE surge to the peripheral arteries leading to apical myocardial stunning, which presents with classical “ballooning” of the cardiac apex [2].

Although the condition is believed to result from stress-induced neurohormonal surge, we present a case of stress-induced cardiomyopathy in a female patient who presented with angina and had ST-segment elevation in leads I and aVL and significant troponin elevation without any preceding stress. She was found to have insignificant coronary artery disease in a coronary angiogram, and moderately reduced left ventricular ejection fraction and regional wall motion abnormalities in the pattern typical of stress-induced cardiomyopathy.

## Case presentation

**Figure 1:**
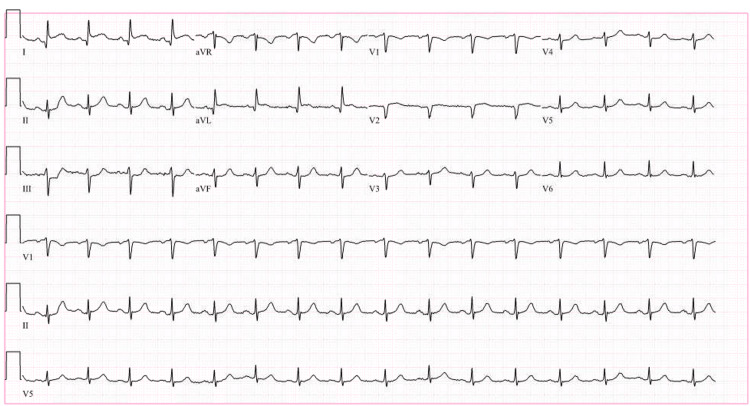
Electrocardiogram showing ST-segment elevation in leads I and aVL

A 72-year-old female with a past medical history of recurrent stage IIA invasive ductal carcinoma of the breast, grade III, estrogen receptor-positive status post bilateral mastectomy, radiation therapy, chemotherapy, and adjuvant endocrine therapy with anastrozole completed a month prior to presentation and on remission presented with anginal chest pain. The patient was hemodynamically stable, and the cardiovascular system examination was unremarkable. ECG showed ST-segment elevation in leads I and aVL (Figure 1) and serial high-sensitivity troponins were elevated at 910 ng/L, 2000 ng/L, and 1400 ng/L.

**Figure 2:**
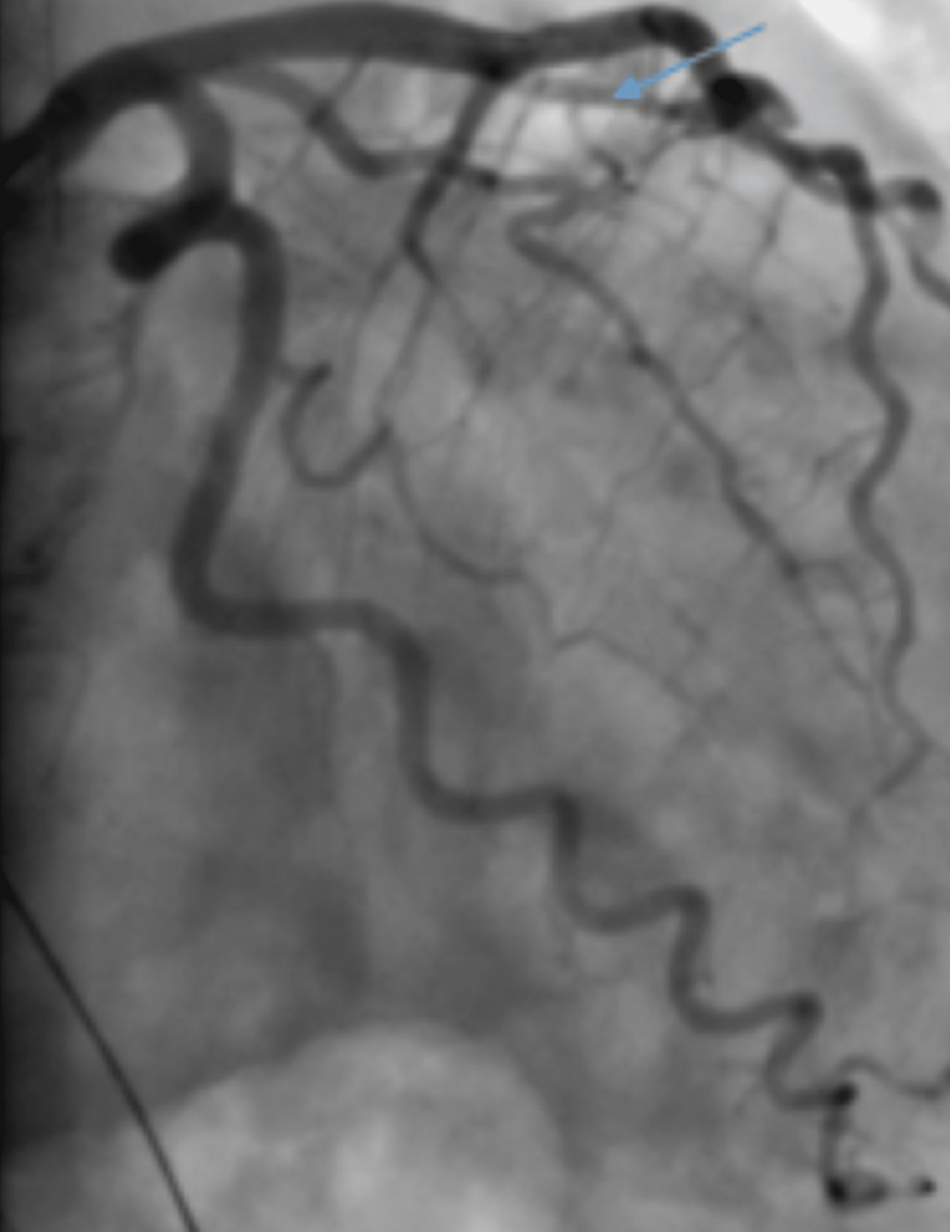
Coronary angiogram RAO caudal view Selective left coronary artery intubation showing a plaque in the proximal D1 (arrow)

She was treated with aspirin 324 mg, clopidogrel 600 mg, and heparin infusion and was emergently taken to the cardiac catheterization lab. A coronary angiogram showed a plaque in the first diagonal artery while other major epicardial coronary arteries were normal (Videos 1-6, Figure 2). 

**Video 1:** Coronary angiogram LAO cranial view Selective left coronary artery intubation showing a plaque in the proximal first diagonal artery (D1) and normal circumflex (CIRC) and left anterior descending coronary artery (LAD) LAO: left anterior oblique

**Video 2:** Coronary angiogram RAO cranial view Selective left coronary intubation showing a plaque in the proximal D1 and normal LAD RAO: right anterior oblique

**Video 3:** Coronary angiogram LAO caudal view Selective left coronary artery intubation showing a plaque in the proximal D1 and normal circumflex and LAD

**Video 4:** Coronary angiogram RAO caudal view Selective left coronary artery intubation showing a plaque in the first diagonal artery and normal circumflex and LAD

**Video 5:** Coronary angiogram LAO cranial view Selective right coronary artery intubation showing a normal proximal, mid, and distal right coronary artery (pRCA, mRCA, and dRCA respectively), right posterior descending artery (RPDA), and right posterolateral branch

**Video 6:** Coronary angiogram RAO cranial view Selective right coronary artery intubation showing a normal proximal, mid, and distal right coronary artery (pRCA, mRCA, and dRCA respectively) and branches

The left ventriculogram showed an ejection fraction of 35%, anteroapical akinesis, and basal hyperkinesis suggestive of stress-induced cardiomyopathy (Video 7).

**Video 7:** Left ventriculogram in RAO cranial projection V-gram showing anteroapical akinesis (A) and hypercontractile base (B)

Transthoracic echocardiography (TTE) showed a moderately reduced ejection fraction of 35% with akinetic mid to distal anterior, lateral walls and apex, and hypokinetic inferior and inferoseptal walls with preserved basal contraction suggestive of stress-induced cardiomyopathy (Videos 8, 9).

**Video 8:** Transthoracic echocardiography: apical two-chamber view Transthoracic echocardiography showing hypokinetic mid to distal inferior and akinetic anterior walls with hypercontractile basal inferior and anterior walls

**Video 9:** Transthoracic echocardiography: apical four-chamber view The view shows a moderately reduced LV ejection fraction of 35% with mid inferoseptal, distal septal, apical, mid, and distal lateral akinesis with basal hyperkinesis suggestive of stress-induced cardiomyopathy

The patient's baseline echocardiography was normal. The wall motion abnormalities along with a moderate reduction in left ventricular systolic function were typical for stress-induced cardiomyopathy and not explained by coronary artery disease. The anginal chest pain, ST-elevation in the ECG in the lateral leads, and significant troponin elevation were due to stress-induced cardiomyopathy. The patient was medically managed with aspirin, metoprolol succinate, atorvastatin, and valsartan and discharged home in stable condition.

## Discussion

Stress-induced cardiomyopathy or Takotsubo cardiomyopathy is the most common manifestation of cardiac neurohormonal stress. Stress-induced cardiomyopathy was first described by Iga et al. as reversible left ventricular dysfunction associated with pheochromocytoma [6]. Sato et al. first described it as Takotsubo-like (octopus trap in Japanese) left ventricular dysfunction in 1990 [7]. Its incidence is on the rise, and patients present with typical chest pain, classical apical hypokinesis, and basal hyperkinesis in echocardiography with elevated cardiac enzymes mimicking ACS and have normal coronary angiography. These patients are treated with beta-blockers, renin-angiotensin-aldosterone system (RAAS) inhibitors with improvement in left ventricular ejection fraction.

SCAD is the other manifestation of myocardial neurohormonal stress and frequently presents with ACS in the same demographic. First described by Pretty in 1931, SCAD is increasingly associated with myocardial infarction (MI) in premenopausal and postmenopausal women without any conventional cardiovascular risk factors [8]; 90% of SCAD patients are women who present with ACS in their fifth and sixth decades of life [9]. SCAD accounts for 35% of MIs in women <50 years and is the most common cause of MI in pregnancy. Studies suggest that SCAD accounts for less than 1% of all MI overall and is very rare among males [10,11]. SCAD has a predilection for the distal vessel, and in terms of coronary distribution, the left anterior descending artery is the most commonly affected artery, and its diagonal and septal branches are involved in 45-61% of cases, followed by the circumflex artery and its branches in about 30% and the right coronary artery and its branches in abound 15% [12]. SCAD is usually diagnosed by invasive coronary angiography; however, it may be easily missed with conventional angiography alone due to its predilection for distal and branch vessels, leading to misdiagnosis as stress-induced cardiomyopathy. Intracoronary imaging modalities like intravascular ultrasound (IVUS) and optical coherence tomography (OCT) have better spatial resolution and should be performed when encountering suspicious lesions if a coronary angiogram is inconclusive [13]. This is important as treatment modalities, prognosis, and follow-up dispositions are different.

## Conclusions

Stress-induced cardiomyopathy may present with chest pain, ischemic ECG changes, significant troponin elevation, and, occasionally, without any evident myocardial stress. It can masquerade as acute MI due to atherosclerotic coronary artery disease in its clinical presentation. It should be differentiated from ACS due to plaque rupture and other sequelae of myocardial stress like SCAD by coronary angiogram as its treatment, outcomes, and prognosis are different.
